# Utilization of Mental Health Care, Treatment Patterns, and Course of Psychosocial Functioning in Northern German Coronary Artery Disease Patients with Depressive and/or Anxiety Disorders

**DOI:** 10.3389/fpsyt.2018.00075

**Published:** 2018-03-12

**Authors:** Anna Lisa Westermair, Anja Schaich, Bastian Willenborg, Christina Willenborg, Stefan Nitsche, Heribert Schunkert, Jeanette Erdmann, Ulrich Schweiger

**Affiliations:** ^1^Department of Psychiatry and Psychotherapy, University of Lübeck, Lübeck, Germany; ^2^Institute for Cardiogenetics, University of Lübeck, Lübeck, Germany; ^3^Partner Site Hamburg and Lübeck and Kiel, German Research Centre for Cardiovascular Research (DZHK), Lübeck, Germany; ^4^University Heart Center Lübeck, Lübeck, Germany; ^5^German Heart Center Munich, Technical University Munich, Munich, Germany; ^6^Partner Site Munich Heart Alliance, German Centre for Cardiovascular Research (DZHK), Munich, Germany

**Keywords:** coronary artery disease, depressive disorders, anxiety disorders, mental health care, utilization behavior, psychosocial functioning, health services research, treatment outcome

## Abstract

**Background:**

Comorbid mental disorders in patients with coronary artery disease (CAD) are common and associated with adverse somatic outcomes. However, data on utilization rates of mental health care and treatment efficiency are scarce and inconsistent, which we tried to remedy with the present preliminary study on Northern German CAD patients.

**Method:**

A total of 514 German CAD patients, as diagnosed by cardiac catheterization, were assessed using the Mini International Neuropsychiatric Interview and the Global Assessment of Functioning (GAF) scale.

**Results:**

Global utilization of mental health care since onset of CAD was 21.0%. Depressive disorders, younger age, and lower GAF at onset of CAD were associated with higher utilization rates, while anxiety disorders and gender were not. Lower GAF at onset of CAD, female gender, and psychotherapy was positively associated with higher gains in GAF, while younger age and anxiety disorders were negatively associated.

**Conclusion:**

The majority of CAD patients with comorbid depression reported to have received mental health treatment and seemed to have benefited from it. However, we found preliminary evidence of insufficiencies in the diagnosis and treatment of anxiety disorders in CAD patients. Further studies, preferably prospective and with representative samples, are needed to corroborate or falsify these findings and explore possible further mediators of health-care utilization by CAD patients such as race, ethnicity, and socioeconomic status.

## Introduction

Comorbid mental disorders, especially depression and anxiety disorders, are common in patients with coronary artery disease (CAD) and are consistently associated with lower quality of life, poorer somatic outcomes, higher mortality, and higher medical costs (1–8). However, contrary to acute coronary syndrome and myocardial infarction (MI), data on the effectiveness of mental health treatment are inconsistent. There is some data on the effectiveness of SSRIs sertraline and citalopram on depressive symptoms in patients with CAD (9, 10), but several studies found no benefit of add-on psychotherapy (PT) such as interpersonal PT or cognitive behavior therapy (10–13).

Although anxiety disorders are as common in CAD patients as depressive disorders (unpublished data), research on their treatment is even scarcer (14). Panic disorder was successfully treated in CAD patients in the small PATCHD trial (15) and additional CBT reduced somatic anxiety in the SUPRIM trial (12, 13). In addition, little is known about the utilization rates of mental health-care services by patients with CAD and comorbid affective or anxiety disorders, as well as about effectiveness of general mental health care in CAD patients outside of the research setting. As the associations between mental health, well-being and CAD have consistently been shown to be moderated by race, ethnicity and socioeconomic factors (16, 17), data from various countries have to be collected to gain a comprehensive overview. Therefore, the aim of this preliminary descriptive study was to provide data on utilization rates of mental health care and treatment efficiency in CAD patients in Northern Germany to inform researchers, local policy makers and developers of mental health programs.

## Materials and Methods

### Participants

This study constitutes a full survey of patients diagnosed with CAD by cardiac catheterization at the University Hospital Schleswig-Holstein in Lübeck, Germany, between February 2004 and December 2012. Being one of only two University Hospitals in the federal state of Schleswig-Holstein, the University Hospital in Lübeck has a big catchment area extending into the neighboring states of Mecklenburg-Vorpommern and Hamburg. A total of 1,182 patients (74.4% male, mean age = 62.2 years, SD = 11.1) gave written informed consent to the use of their data and to be contacted for research purposes, in accordance with the Declaration of Helsinki. The study protocol was approved by the ethics committee of the University of Lübeck (ID 04/041). When contacted between March 2013 and January 2015, 511 patients agreed to participate in the telephone survey described in this paper. Exclusion criteria were a lack of informed consent, exclusion of CAD during cardiac catheterization and inability to participate (e.g., due to hearing impairment, aphasia, cognitive deficits, or insufficient language skills).

### Assessment

#### Onset of CAD

Onset of CAD was defined as the year in which one of the following criteria was met for the first time: (a) specific symptoms of CAD (e.g., angina pectoris) not otherwise explicable, (b) cardiac catheterization showing CAD or a diminished cardiac function, and (c) formal diagnosis of MI.

#### Anxiety and Depressive Disorders

Participants were assessed for mental disorders with the Mini International Neuropsychiatric Interview (M.I.N.I. 5.0.0), a short, structured diagnostic interview for DSM-IV (18) and ICD-10 (19) psychiatric disorders with good psychometric qualities. The interview was conducted with regard to the years after onset of the CAD. Mental disorders were grouped in depressive disorders (Major Depressive Episode and Dysthymia) and anxiety disorders (Agoraphobia, Panic Disorder, Social Phobia, Generalized Anxiety Disorder, Posttraumatic Stress Disorder, Obsessive Compulsive Disorder, and Hypochondria). Other severe mental disorders were assessed but excluded from further analyses due to low frequency: of the participants who did not meet criteria for a depressive or anxiety disorder, only eight met criteria for another severe mental disorder, e.g., bipolar, substance related, eating or somatoform disorder. Mild and moderate depressive episodes as well as adjustment disorders were not assessed.

#### Psychosocial Functioning

The Global Assessment of Functioning (GAF) of the DSM-IV was used to estimate psychosocial functioning on a scale from 1 to 100 (e.g., 1–10: severe impairment; 90–100 superior functioning) (20). This standard method of judgment of a participant’s current level of psychological, social, and occupational functioning has shown good psychometric properties (21, 22).

#### Utilization of Mental Health Care

As this was a preliminary, fact-finding study, we operationalized mental health care very broadly as any care by a certified professional meant to alleviate mental troubles. Accordingly, participants were asked if they had ever consulted a psychiatrist, been prescribed a drug for mental troubles, underwent outpatient PT (>4 sessions) or been hospitalized for mental disorders. In the case of an answer in the affirmative, timing of mental health care was assessed with regard to onset of the CAD. We did not collect data on the identities of the mental health professionals that provided care for the participants of our study. Of note, one particularity of the German mental health-care system is a high rate of voluntary hospitalizations for dosing of antidepressants and/or inpatient PT. None of the participants of our study reported an *involuntary* admission to a psychiatric hospital.

### Procedure

Trained and regularly supervised interviewers of the psychological and medical staff of the University Hospital Schleswig-Holstein, Lübeck, Germany, contacted participants to inform them about the content and purpose of the survey. When consent was renewed, onset of CAD was determined, and the M.I.N.I. was conducted with regard to years before and after the onset of CAD as well as to the present. After scoring the GAF, the interviewers informed participants about access to treatment (if necessary). In the example of acute suicidality, which did not occur, study protocol specified alerting the psychiatrist on duty. At the end of the interview, participants were offered a summary of the research findings in layman’s terms upon completion of the study, in which case contact details were noted.

### Statistical Analysis

SPSS 23 for Windows (SPSS Inc., USA) was used for all statistical analyses. To test for influence of the length of the interval between onset of CAD and the telephone interview on the prevalence rates of mental disorders and the utilization rates of mental health care, we performed point-biserial correlations. In χ^2^ tests, Cramér’s *V* was used to measure effect size, and *z*-test and standardized residuals to breakdown significant χ^2^ tests. Interactions in ANOVA were followed-up with *t*-tests and Games–Howell-*post hoc* tests as appropriate. To compensate for violations of assumptions regarding sampling distributions, resampling was applied where necessary using bias corrected and accelerated (BCa) bootstrapping with a significance level of 0.05. Due to the exploratory nature of the present study, we refrained from correction for multiple comparisons to prevent accumulation of β error.

## Results

### Sample Characteristics

Of the 1,182 participants who were contacted for the telephone survey, 269 could not be reached, 207 were known to be deceased, 138 declined consent, 26 suffered from communication problems such as insufficient language skills or hardness of hearing, 18 could not participate due to impairment of cognitive functions, and 13 participants were excluded for other reasons. Thus, response rate was 43.2%. The final sample consisted of 511 participants (75.9% male, mean age = 60.1 years, SD = 10.11). Mean onset of CAD was at 55.3 years (SD = 10.8).

### Psychiatric Morbidity

In the years between CAD onset and the telephone interview (M = 10.9 years, SD = 8.2), 10.5% of participants met the criteria for an anxiety disorder, 11.3% for a depressive disorder, and 6.0% for both an anxiety and depressive disorder. 72.2% did not meet criteria for any depressive or anxiety disorder (unpublished data). These prevalence rates did not correlate with the length of the interval between CAD onset and the telephone interview (all *r*s ε [−0.05; 0.05], all *p*s ≥ 0.180).

### Utilization of Mental Health Treatment

The global utilization rate of mental health care was 20.1% and was highest in participants with comorbid depressive and anxiety disorders (67.7%, see also Table 1), followed by participants with depressive disorders (53.7%). In comparison, participants with anxiety disorders (22.4%) and participants with neither depressive nor anxiety disorders (12.1%) were significantly less likely to have received mental health care [χ^2^(3) = 93.27, *p* < 0.001]. Utilization of all types of mental health treatment (psychopharmacologic, psychotherapeutic, or combination) was highest in participants with depressive disorders, regardless of comorbidity with anxiety disorders [all χ^2^(3) ≥ 24.9, *p* < 0.001]. The same overall utilization pattern emerged regarding the utilization of outpatient mental health treatment at the time of the interview, only with lower frequencies (McNemar *p* < 0.001). None of the rates of utilization of mental health care correlated with the length of the interval between CAD onset and the telephone interview (all *r*s ε [−0.08; 0.07], all *p*s ≥ 0.073).

**Table 1:** Frequencies of utilization of different types of mental health care.

			After						Current					
	
			Total	Neither depressive nor anxiety disorder	Only anxiety disorder	Only depressive disorder	Both depressive and anxiety disorder		Total	Neither depressive nor anxiety disorder	Only anxiety disorder	Only depressive disorder	Both depressive and anxiety disorder	
	
		*n*	514	371	58	54	31		514	421	53	25	15	

			(%)					χ^2^	(%)					χ^2^
Type of mental health treatment	Outpatient	Psychiatric consultation	16.1	7.8	19.0	44.4	61.3	98.0*	6.8	3.8	9.4	32.0	40.0	57.6*
		Standardized residuals		−4.0	0.5	5.2	6.3			−2.4	0.7	4.8	4.9	

		Psychopharmacotherapy	9.7	3.2	10.3	31.5	48.4	99.7*	6.6	4.5	7.5	24.0	33.3	32.7*
		Standardized residuals		−4.0	0.2	5.1	6.9			−1.7	0.3	3.4	4.0	

		Psychotherapy	9.3	2.7	13.8	33.3	38.7	89.0*	3.1	1.2	3.8	24.0	20.0	55.6*
		Standardized residuals		−4.2	1.1	5.8	5.4			−2.2	0.3	5.9	3.7	

		Any outpatient mental health treatment	21.0	12.1	22.4	53.7	67.7	93.3*	8.9	5.7	11.3	36.0	46.7	54.5*
		Standardized residuals		−3.7	0.2	5.2	5.7			−2.2	0.6	4.5	4.9	

	Inpatient	Any inpatient mental health treatment	4.1	1.3	5.2	14.8	16.1	34.6*	0.6	0.0	0.0	8.0	6.7	36.1*
		Standardized residuals		−2.6	0.4	3.9	3.3			−1.6	−0.6	4.9	3.1	

	Any	Any mental health treatment	21.4	12.4	24.1	53.7	67.7	91.2*	8.9	5.7	11.3	36.0	46.7	54.5*
		Standardized residuals		−3.7	0.5	5.1	5.6			−2.2	0.6	4.5	4.9	

*After = relative frequency of respective type of mental health care in the period after onset of the coronary artery disease. Current = relative frequency at the time of the interview. χ^2^ = chi square test statistic for the comparison between the four diagnostic subgroups (neither depressive nor anxiety disorder, only anxiety disorder, only depressive disorder, both depressive and anxiety disorder)*.

*Significant at the 0.05 level.

Utilization of inpatient mental health treatment was highest in participants with comorbid depressive and anxiety disorders (16.1%), followed by participants with depressive disorders (14.8%). Participants with anxiety disorders (5.2%) and participants with neither depressive nor anxiety disorders (1.3%) again were significantly less likely to have received inpatient mental health care [χ^2^(3) = 34.61, *p* < 0.001]. Overall utilization of inpatient mental health treatment was lower (McNemar’s *p* < 0.001) with only participants with depressive disorders (with or without comorbid anxiety disorders, 3.7 or 3.2%, respectively) currently in inpatient mental health care.

Psychopharmacotherapy without specialist (namely, psychiatric) consultation was uncommon (22% of participants receiving medication) and occurred mainly among participants meeting criteria for neither depressive nor anxiety disorders (61.5%).

There was no significant gender difference in the global utilization of mental health care or for any specific type of mental health treatment.

Binary logistic regression was performed to identify predictors of receiving mental health care after onset of CAD. A model including age at onset of CAD, gender, utilization of mental health treatment before onset of CAD, GAF after onset of CAD, depressive disorders and anxiety disorders after onset of CAD was a significant fit for the data [χ^2^(6) = 117.70, *p* < 0.001, *R*^2^ = 0.22 (Cox and Snell), =0.33 (Nagelkerke’s), Hosmer–Lemeshow Test: χ^2^(8) = 6.69, *p* = 0.57]. Participants with younger age at onset of CAD as well as low GAF after onset of CAD were more likely to receive any kind of mental health treatment [Wald (1) = 5.94, *p* = 0.02; Wald (1) = 27.36, *p* < 0.001]. Participants with depressive disorder were 2.7 times more likely to receive any kind of mental health treatment [Wald (1) = 8.1, *p* = 0.004], whereas meeting criteria for an anxiety disorder did not significantly predict the utilization of mental health care (OR = 1.3). Likewise, neither gender nor previous utilization of mental health treatment was predictors of utilization of mental health treatment after onset of the CAD.

Participants with depressive disorders were as likely to receive psychopharmacotherapy (37.6%) as PT (35.3%; McNemar’s χ^2^ = 0.04, *p* = 0.85).

### Interaction between Treatment Patterns and Course of Psychosocial Functioning

To quantify proxy markers of effectiveness of mental health treatment in CAD patients, we conducted an ANCOVA with gender, depressive disorder, anxiety disorder, medication, and PT (all after onset of the CAD) as fixed factors, age at onset and lowest GAF after onset of the CAD as covariates and difference between lowest GAF after onset of CAD and GAF at the time of the interview (Δ GAF) as criterion. There was a main effect for age [*F*(1, 450) = 8.38, *p* = 0.004, partial η^2^ = 0.02] with participants younger than 55 years of age at onset of their CAD gaining 3.78 GAF points fewer than older participants [*T*(547.10) = 3.96, BCa 95% CI = [1.95; 5.66], *p* = 0.001].

There was a main effect for minimum GAF after onset of the CAD [*F*(1, 450) = 90.64, *p* < 0.001, partial η^2^ = 0.17]. Participants whose GAF sank below 70 after onset of their CAD gained on average 9.63 GAF points more than those whose GAF never fell below 70 [*T*(264.79) = 9.96, BCa 95% CI = [7.84; 11.56], *p* = 0.001].

There was a main effect for gender [*F*(1, 450) = 5.42, *p* = 0.020, partial η^2^ = 0.01], with women gaining 5.1 GAF points (SE = 2.0) more than men.

There was a main effect for anxiety disorders [*F*(3, 450) = 8.48, *p* = 0.004, partial η^2^ = 0.02]. Participants with anxiety disorders gained 7.8 GAF points less than those without (SE = 1.9, *p* < 0.001). There was no main effect for depressive disorders [*F*(1, 450) = 0.31, *p* = 0.577, Δ GAF gain = 0.92 points].

There was a main effect for PT [*F*(3, 450) = 7.77, *p* = 0.006, partial η^2^ = 0.02]. Participants who underwent PT after onset of their CAD gained 5.51 GAF points more than those who did not (SE = 1.97, *p* = 0.005). This pattern held true when only subjects without depressive or anxiety disorders were included in the analysis [*F*(1, 336) = 17.81, *p* < 0.001, partial η^2^ = 0.05, Δ GAF gain = 16.16 points, BCa 95% SE = 2.33, CI = [10.43; 20.35], *p* < 0.001].

**Figure 1:**
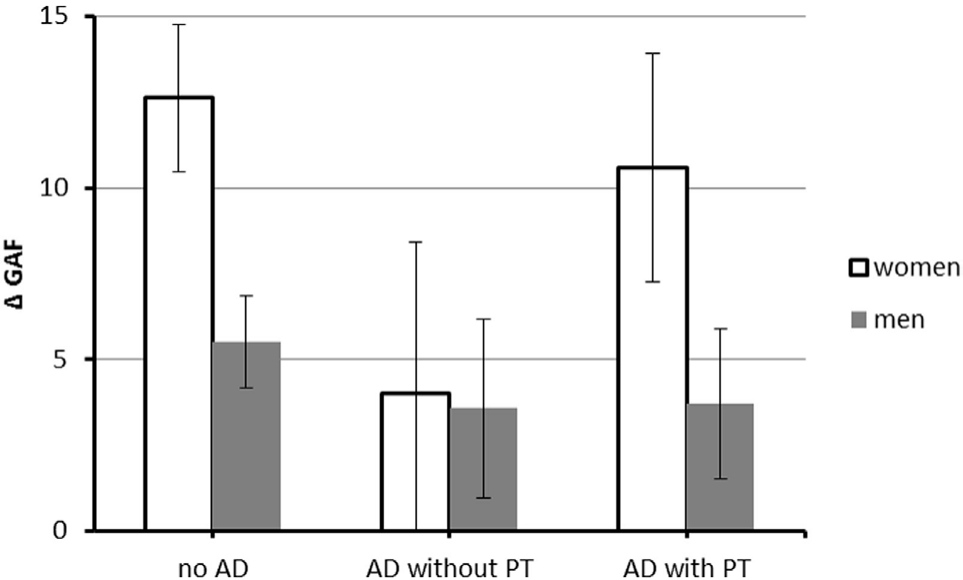
Interaction between anxiety disorders, PT, and gender on difference between minimum GAF after onset of coronary artery disease (CAD) and GAF at interview. *N* = 481 (117 women and 364 men). Abbreviations: GAF, Global Assessment of Functioning; AD, anxiety disorder; PT, psychotherapy. Values are corrected for the effect of covariates [age at onset of CAD, minimum GAF after onset of CAD]. Error bars indicate SE.

There was a three-way interaction between PT, anxiety disorders (AD), and gender [*F*(3, 450) = 8.05, *p* = 0.005, partial η^2^ = 0.02, see also Figure 1]. In women with anxiety disorders, PT was associated with higher GAF gain (M_AD with PT_ = 10.58, SE = 4.06 vs. M_AD without PT_ = 4.02, SE = 3.31), but this was not true in men (M_AD with PT_ = 3.71, SE = 2.46 vs. M_AD without PT_ = 3.57, SE = 2.27).

There was no main effect for medication [*F*(3, 450) = 0.10, *p* = 0.756, Δ GAF gain = 2.59 points], but a two-way medication × depressive disorders interaction was found [*F*(3, 450) = 6.10, *p* = 0.014, partial η^2^ = 0.01]; in participants with depressive disorders, medication was associated with higher GAF gain (Δ GAF gain = 7.53, BCa 95% SE 3.71, *p* = 0.040, CI = [0.28; 14.74]).

**Figure 2:**
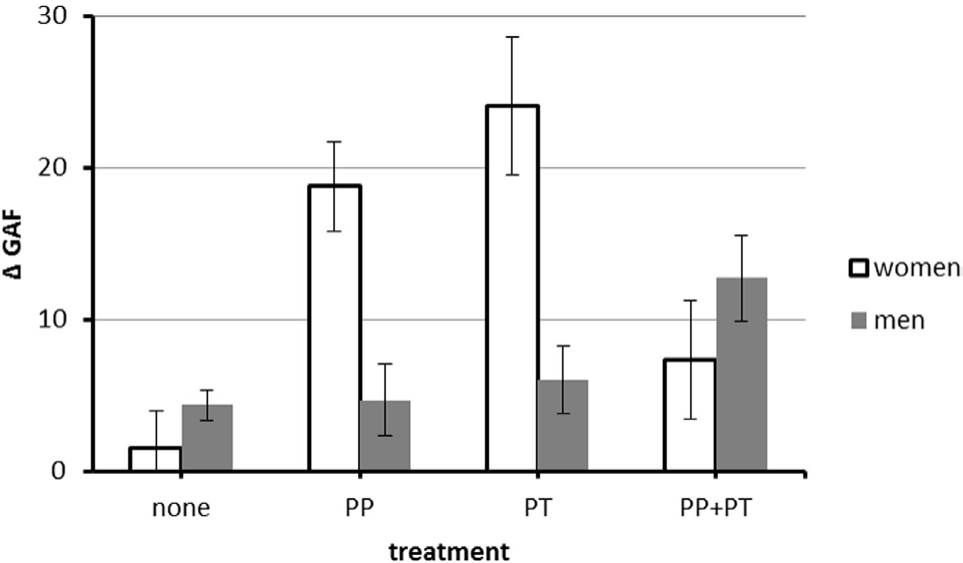
Interaction between PT, medication, and gender on difference between minimum GAF after onset of the coronary artery disease (CAD) and GAF at the interview. *N* = 481 (117 women and 364 men). Abbreviations: GAF, Global Assessment of Functioning; PP, psychopharmacotherapy; PT, psychotherapy. Values are corrected for the effect of covariates (age at onset of CAD, minimum GAF after onset of CAD). Error bars indicate SE.

There was an interaction between PT and medication [*F*(3, 450) = 8.15, *p* = 0.004, partial η^2^ = 0.02]. PT and combination treatment were associated with higher GAF gain than no treatment (both M ≥ 11.24, both Games–Howell *p*s < 0.001), but no type of treatment was superior to another (all *p*s ≥ 0.48). There was also a three-way interaction between PT, medication, and gender [*F*(3, 450) = 22.05, *p* < 0.000, partial η^2^ = 0.05, see also Figure 2]. In women, PT (PT) was associated with the highest GAF gain (M_PT_ = 24.07, SE 4.54 vs. M_MED_ = 18.79, SE = 2.94 and M_COMB_ = 7.34, SE 3.89), whereas in men, combination therapy (COMB) was associated with the highest GAF gain (M_COMB_ = 12.74, SE = 2.81 vs. M_MED_ = 4.72, SE = 2.34 and M_PT_ = 6.06, SE 2.24).

Re-running this model with the length of the interval between onset of CAD and the telephone interview as additional covariate did not yield qualitatively different results and there was no main effect for the length of the interval [*F*(1, 449) = 0.38, *p* = 0.54, partial η^2^ = 0.00].

## Discussion

In our sample, one in five participants had received mental health care after onset of their CAD, and one in two participants met criteria for an anxiety and/or depressive disorder. Apart from diagnoses, other predictors of mental health-care utilization were young age at onset of the CAD and low psychosocial functioning after onset of the CAD. PT was associated with higher gains in psychosocial functioning in all diagnostic groups, whereas medication was associated with higher gains only in participants with depressive disorders. On the contrary, male gender and anxiety disorders were associated with fewer gains in psychosocial functioning.

The utilization rates of general mental health care in our study of CAD patients were comparable to data from the respective age cohort of the general German population (23, 24), to data from a nationally representative sample of elderly persons with depressive symptoms and chronic somatic diseases (25) and to data from another chronically somatically ill sample in Germany, cancer patients (26). Some participants reported previous mental health treatment without meeting criteria for a depressive or anxiety disorder. They possibly suffered from mild mental disorders such as adjustment disorder (not taken into account in this study) or were prescribed medication in absence of a mental disorder (27–29).

Younger participants and participants with greater psychosocial impairment were more likely to seek mental health care, which is in agreement with data from the German general population (23) and U.S. patients with chronic somatic diseases and depression (30). Higher utilization of mental health care by younger patients may be attributable to higher distress (due to more severe practical consequences of psychological impairment, e.g., at work) or fewer barriers to seeking mental health treatment: younger CAD patients may have greater knowledge of mental health and be less prone to self-stigmatization.

One in two participants with a depressive episode reported to have received any mental health treatment (e.g., psychopharmacotherapy, PT, or a combination of both), which is comparable to data from the Medical Expenditure Panel Survey on depressed CAD patients in the U.S. (30). However, the patterns of treatment modality were strikingly different. Whereas participants in our study were as likely to receive PT as psychopharmacotherapy, the vast majority of participants in the U.S. received psychopharmacotherapy. This finding probably reflects differences in health insurance: for the vast majority of residents, costs for PT are reimbursed by German statutory health insurance. Therefore, roughly 10% of the German general population consulted a psychologist or psychotherapist in 2014, nearly twice the European average (31).

Participants with anxiety disorders were less likely to receive any mental health care than participants with depressive disorders, which is surprising as anxious cardiac patients are known to consult their general practitioners more frequently (32, 33). We hypothesize that patients and/or GPs have trouble recognizing anxiety disorders, which could be ameliorated by specific public education or psychological liaison services in cardiology.

Also, mental health care was not associated with higher gains in psychosocial functioning in participants with anxiety disorders. This finding may be due to a variety of reasons. Efficacy of psychopharmacotherapy for anxiety in CAD patients is uncertain as there is no evidence to date. Evidence-based PT is also not available as standard CBT for anxiety disorders (including exposure therapy) can have adverse somatic effects in CAD patients (34–37), and no adaptation for this patient population has yet been implemented. However, the PATCHD trial recently proved feasibility of such an adaption (15).

Surprisingly, in women, the combination of psychopharmacotherapy and PT was not associated with higher GAF gains compared with psychopharmacotherapy or PT alone.

A strength of the present study is its setting in routine health care as opposed to research centers, pointing to high ecologic validity of the results. The sample is also large and representative for Northern Germany. A high level of training for the interviewers, including regular supervision as well as assessment with two intensely validated, semi-structured interviews, the MINI and the GAF, allow for the assumption of good reliability of the findings.

However, our study is based on a convenience sample, which limits generalizability of the findings. The retrospective study design could have led to underestimation of utilization rates due to compromised recall and clearly reduced the reliability of GAF ratings. Using self-reported onset of CAD as a reference point without standardization of the time point of contacting by the study team led to different survey periods for each participant, which complicated the comparison of our findings with existing data on utilization rates of mental health care (while improving ecological validity). The assessment by telephone may have led to misunderstandings due to lack of nonverbal communication (reducing sensitivity) and selection bias, as critically ill and/or hospitalized patients are less likely to answer the telephone. Our operationalization of mental health care also did not consider length of treatment, increasing the risk of overestimation of participants receiving mental health treatment.

### Outlook

As the associations between mental health, well-being and CAD have consistently been shown to be moderated by race, ethnicity and socioeconomic factors (16, 17), future studies should compare mental health-care utilization rates of CAD patients across countries and social environments.

### Conclusion

To the best of our knowledge, this is the first study on utilization rates of mental health care in CAD patients in Northern Germany. One in five of all participants in our preliminary study and one in two participants who met criteria for major mental disorder reported to have received mental health care after onset of their CAD. The majority of CAD patients with comorbid depression reported to have received mental health treatment and seemed to have benefited from it. However, we found preliminary evidence for insufficiencies in the diagnosis and treatment of anxiety disorders in CAD patients in Germany. Further studies, preferably prospective and with representative samples, are needed to corroborate or falsify these findings and explore possible further mediators of health-care utilization by CAD patients such as race, ethnicity, and socioeconomic status.

## Ethics Statement

This study was carried out in accordance with the recommendations of the ethics committee of the University of Lübeck, Germany, with written informed consent from all subjects. All subjects gave written informed consent in accordance with the Declaration of Helsinki. The protocol was approved by the ethics committee of the University of Lübeck, Germany (ID 04/041).

## Author Contributions

BW, CW, HS, JE, and US contributed to the conception and design of the study; AW, AS, BW, and SN acquired the data and organized the database; AW, AS, and US performed the statistical analysis; AW and AS wrote the first draft of the manuscript. All the authors contributed to manuscript revision, read and approved the submitted version.

## Conflict of Interest Statement

The authors declare that the research was conducted in the absence of any commercial or financial relationships that could be construed as a potential conflict of interest.
